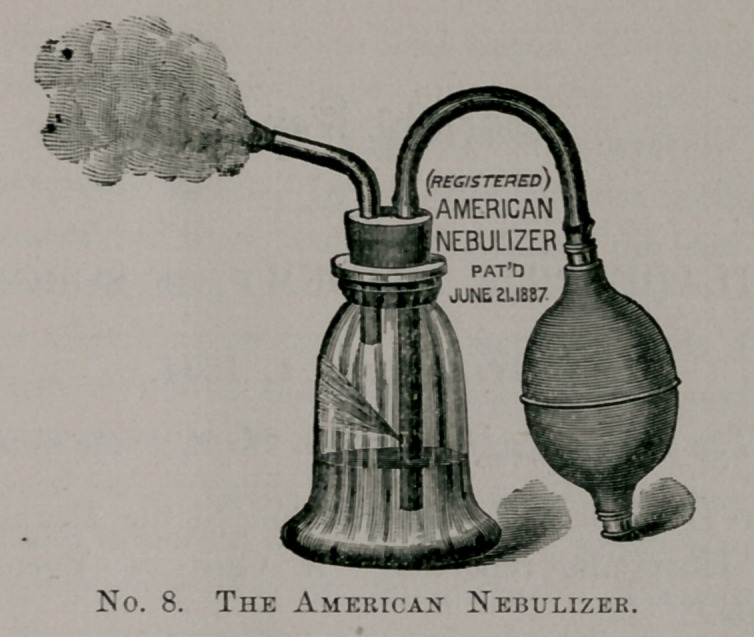# Nasal Sprays

**Published:** 1894-08

**Authors:** George Brown

**Affiliations:** Atlanta, Ga.


### NASAL SPRAYS. WHAT APPARATUS TO USE.

BY GEORGE BROWN, M.D., ATLANTA, GA.

I wish to present to the general practitioner, who, in the hurry of a practice that does not enable him to keep as well posted on such matters as he would probably wish to, a few of the most modern and serviceable sprays.

For spraying oils, where one wishes to use them warmed, unquestionably the best spray is the one shown in the cut below—De Vilbiss, No. 9. I prefer this pattern to his others. Both of his makes can be used for nose, throat and post-nasal space by the patients themselves, with a little practice. It is all metal, easily cleaned and will last a lifetime.

No. 1. DE VILBISS SPRAY, No. 9.

The Davidson, No. 159 is probably the next best and strongest made spray for throwing oils. This has extra tips for the throat and is very strong and has the advantage of throwing an almost continuous spray.

No. 2. THE DAVIDSON RUBBER CO. SPRAY, No. 159.

This firm also makes a nasal spray, No. 548, that is very compact and can easily be carried in a very small space, making it very convenient for traveling men, etc. The top can be taken off and the tubes easily cleaned.

THE DAVIDSON ATOMIZER, No. 548.

Messrs. Codman & Shurtleff make one very similar to the above of which an immense number have been sold. Either of these are

No. 4. CODMAN & SHURTLEFF ATOMIZER.

very good for nasal sprays, but cannot be of service to a great many cases where there is a diseased condition of the post-nasal space. In these cases it is better to use an apparatus that can be introduced behind the soft palate.

The same firm make the apparatus in the cut below. This is somewhat similar to the Davidson, No. 159, and can be used for both throat and post-nasal space, as well as for the nose. This throws an almost continuous spray, and is an instrument that will give satisfaction.

No. 5. CODMAN & SHURTLEFF NOSE AND THROAT ATOMIZER.

Messrs. Chas. Truax, Green & Co. make the instrument shown below. They also make a preparation called "Camenthol," which

No. 6. THE TRUAX-GREEN ATOMIZER.

they advertise in connection with the instrument, though the instrument will spray any of the oily preparations very nicely. It is most useful when the trouble is confined to the nose.

E. B. Meyrowitz makes two sizes of Dr. Leffert's sprays which are very fine for use in the nose and throat. They come nearer throwing a continuous spray than any hand spray I have ever seen.

The S. H. Wentmore Company make two sizes of the "Century" Sprays. They are No. 5 and No. 15. I have prescribed quite a lot of each, and they give good satisfaction.

For inhalations I use three kinds of sprays. Unquestionably the best is the apparatus of Dr. H. S. Dunlap, of Battle Creek, Mich., which is called "The Globe Nebulizer." I do not think this instrument can be improved upon, especially when used as I use it in my office, connected to a compressed air apparatus carrying twenty-five pounds pressure. I am getting better results from the use of this instrument in middle ear troubles than anything I have ever used.

No. 7. THE GLOBE ATOMIZER.

E. B. Meyrowitz makes the American Nebulizer, which is a cheaper apparatus than the above, but is very good for patient's home use. I am prescribing these with good results. The fluid is broken into very fine particles and can be inhaled with absolute ease.

No. 8. THE AMERICAN NEBULIZER.

Messrs. Codman & Shurtleff also make a splendid instrument for the same purpose. It is one of the best I know of, and I have my druggist to keep it in stock for me also. It is of glass and will last a lifetime, and is also a cheaper instrument than the Globe.

For spraying peroxide of hydrogen or pyrozone, Messrs. McKesson & Robbins make the prettiest and most useful of sprays. No one, who pretends to treat any trouble of the upper air passages, can afford to be without their set of sprays. While not a spray, I deem it my duty to call your attention to their "Pulverflator" for applying the stearate of zinc preparations to the upper air passages. With this instrument and a full line of their preparations of the stearates and combinations, much relief can be afforded cases that cannot be reached in any other way.

The question of what to use in the different diseased conditions of the nasal passages is too broad to be touched upon, and I can only say that there is no preparation that one would wish to use in the nose that cannot be applied by some one of the instruments described above.

## Figures and Tables

**No. 1. f1:**
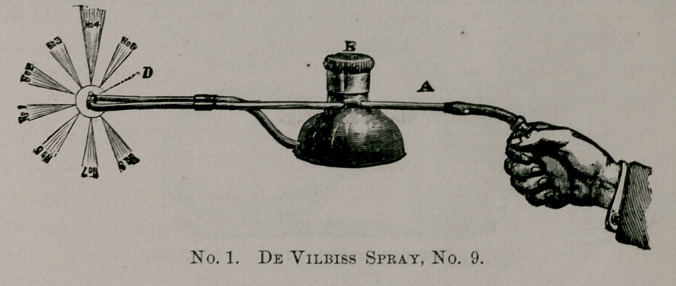


**No. 2. f2:**
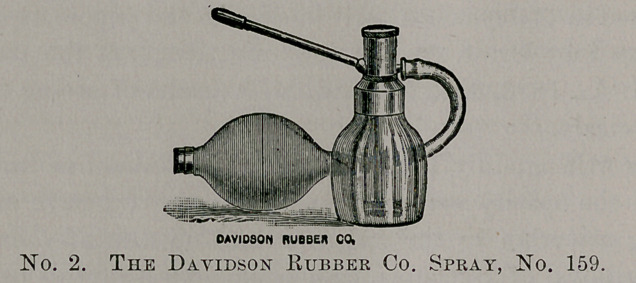


**Figure f3:**
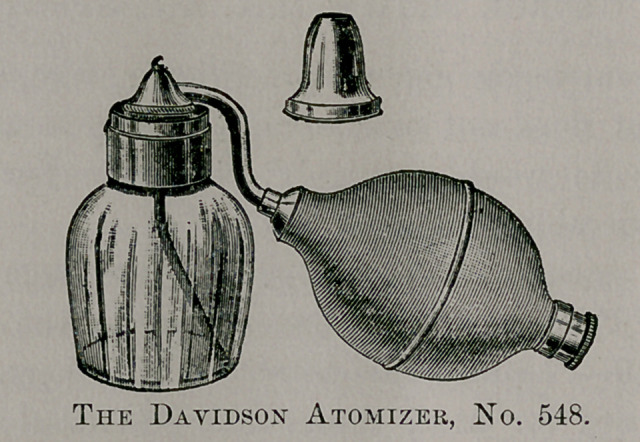


**No. 4. f4:**
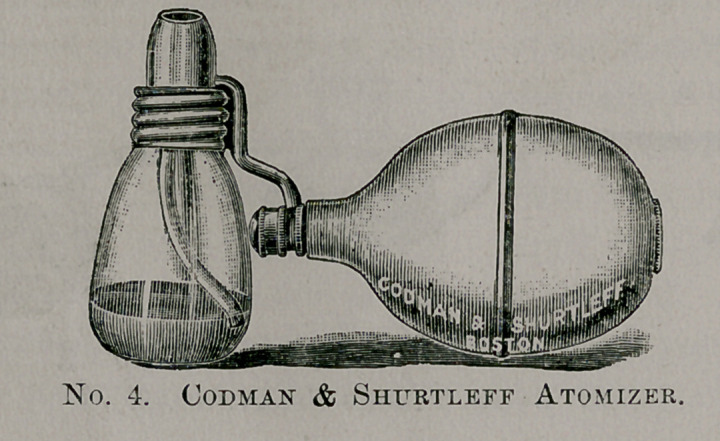


**No. 5. f5:**
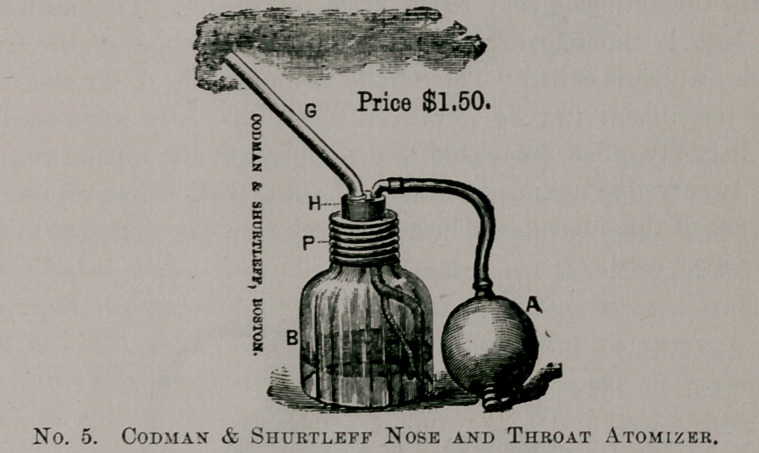


**No. 6. f6:**
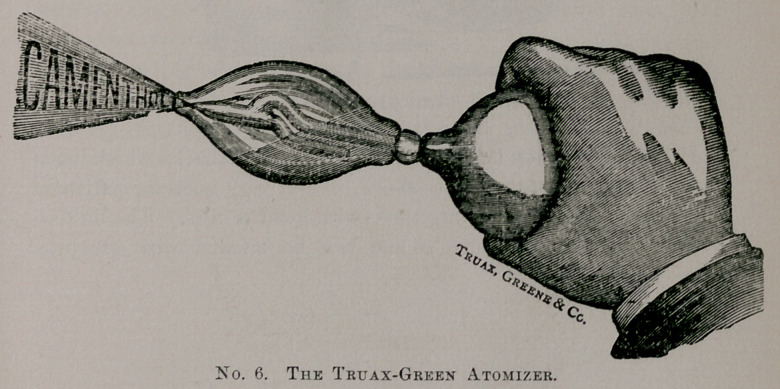


**No. 7. f7:**
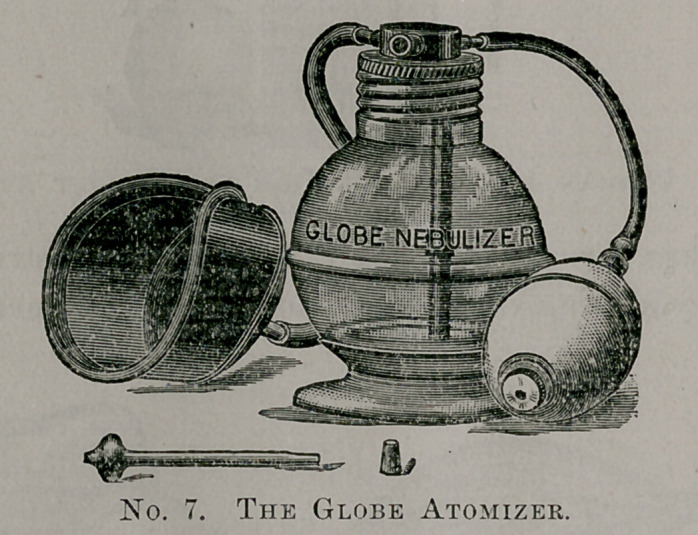


**No. 8. f8:**